# Innovative Design of Residual Stress and Strain Distributions for Analyzing the Hydrogen Embrittlement Phenomenon in Metallic Materials

**DOI:** 10.3390/ma15249063

**Published:** 2022-12-19

**Authors:** Jesús Toribio, Miguel Lorenzo, Leticia Aguado

**Affiliations:** Fracture & Structural Integrity Research Group, University of Salamanca, E.P.S., Campus Viriato, Avda. Requejo 33, 49002 Zamora, Spain

**Keywords:** pearlitic steel, prestressing steel, wire drawing, residual stresses, notch, finite elements, hydrogen embrittlement (HE), “*a-la-carte*” residual stresses

## Abstract

Round-notched samples are commonly used for testing the susceptibility to hydrogen embrittlement (HE) of metallic materials. Hydrogen diffusion is influenced by the stress and strain states generated during testing. This state causes hydrogen-assisted micro-damage leading to failure that is due to HE. In this study, it is assumed that hydrogen diffusion can be *controlled* by modifying such residual stress and strain fields. Thus, the selection of the notch geometry to be used in the experiments becomes a key task. In this paper, different HE behaviors are analyzed in terms of the stress and strain fields obtained under diverse loading conditions (un-preloaded and preloaded causing residual stress and strains) in different notch geometries (shallow notches and deep notches). To achieve this goal, two uncoupled finite element (FE) simulations were carried out: (i) a simulation by FE of the loading sequences applied in the notched geometries for revealing the stress and strain states and (ii) a simulation of hydrogen diffusion assisted by stress and strain, for estimating the hydrogen distributions. According to results, hydrogen accumulation in shallow notches is heavily localized close to the wire surface, whereas for deep notches, hydrogen is more uniformly distributed. The residual stress and plastic strains generated by the applied preload localize maximum hydrogen concentration at deeper points than un-preloaded cases. As results, four different scenarios are established for estimating “*a la carte*” the HE susceptibility of pearlitic steels just combining two notch depths and the residual stress and strain caused by a preload.

## 1. Introduction

High-strength pearlitic steel wires are highly susceptible to hydrogen embrittlement (HE) fracture phenomena [1]. This way, HE is considered the main cause of in-service failure of different mechanical components [2] and prestressing steel wires in the presence of aggressive environments [3,4]. Briefly, HE is developed in a sequence of well-known stages [5,6]: (i) molecular hydrogen adsorption in the material surface from the harsh environment, (ii) dissociation of the hydrogen molecule, (iii) atomic hydrogen absorption into the material, (iv) hydrogen diffusion through the material lattice toward the prospective fracture zones [7] where hydrogen is accumulated [8] up until it reaches a critical concentration linked with the hydrogen micro-damage. In a recent study, Gong et al. analyzed the HE mechanisms in high-strength steels [9]. Texture and microtexture are influencing factors in HE transport toward the zone of hydrogen entry, as it was revealed in the study [10]. Hydrogen permeation was studied in [11] for three different pipeline steels to reveal the HE susceptibility and, in [12], the hydrogen permeation was numerical simulated to reveal the hydrogen apparent solubility and hydrogen trapping site characteristics: trapping density and hydrogen-trapping binding energy.

The key stage that governs HE is the hydrogen diffusion through the material lattice. This process is strongly dependent on the stress and strain state as it was revealed in previous studies [13,14]. Thus, many studies [13,15] are focused on hydrogen diffusion assisted by stress and strain as a way of estimating the HE susceptibility of steels. This way, results obtained from such an analysis reveal the prospective places where hydrogen micro-damage could take place, the so-called *tearing topography surface* (TTS). This term was first coined by Costa and Thompson [16,17] and lately studied by Toribio et al., revealing the characteristics of the TTS [18] as the zone associated with HE in pearlitic steels [19]. In a previous study [20], an analysis of TTS from the fracture mechanics approach was carried out. Furthermore, the evolution of the microstructural damage in pearlitic steel was studied in [21]. Triaxialty is a key factor in the matter of hydrogen-assisted micro-damage (HAMD), as it was pointed out in [22]. The variation of triaxiality with time influences the evolution of HAMD, as it was shown in [23].

The International Federation for Prestressing (FIP) has established a testing method for estimating the HE susceptibility of steels, the so-called FIP test [24]. Essentially, this test consists of applying to a steel sample a constant load (80% of the fracture load in air of such a sample) in the presence of an aggressive environment (20% aqueous solution of ammonium thiocyanate (NH_4_SCN)). The result of a FIP test is the time up to the final fracture of the sample. Although this method is currently used for the acceptance of steels, it exhibits a key shortcoming: the high scattering in results that can be linked to variations of the surface roughness or the existence of residual stress [25]. Thus, the hydrogen entry into the steel (one of the stages of HE, hydrogen adsorption and absorption) is very sensible to material surface roughness and, on the other hand, the inward gradient of manufacturing-induced residual stress acts as a driving force for hydrogen diffusion according to the hydrogen diffusion model assisted by stress and strain [15]. 

Alternative test methods for the estimation of HE are the so-called *slow strain rate test* (SSRT) or *constant extension rate tensile* (CERT) tests [26]. This type of test consists of applying a constant extension rate in an aggressive environment until final failure [27]. The HE susceptibility is obtained by comparing the measurements taken during and after the test with similar data obtained in an inert environment [27]. The crosshead speed influence was studied in [28] and the influence of the loading rate on HAMD was analyzed in [29]. In recent times, diverse test methods for evaluating HE susceptibility were considered. This way, Matsumoto et al. analyzed hydrogen behavior in high-strength steels corresponding to diverse HE testing methods [30]. A test procedure for evaluating the HE of tempered martensitic steels with intergranular fractures was studied in [31]. An in situ fracture experiment for revealing HE was proposed in [32]. 

CERT tests (proposed in the international standard ISO 7539-7 [26]) are widely applied as a suitable method for evaluating the HE susceptibility of high-strength steels [27]. According to the aforesaid standard, HE tests can be developed using different types of samples (slim [27], notched [33], single-edge notch [34], side-grooved [35], and precracked [36,37]). However, the circumferentially notched samples exhibit key advantages that make them suitable for this type of test [38]. In the study [39], the competition of hydrostatic stress and plastic strain on HE in rounded notches and cracks was studied. A fracture criterion for the notch strength in the presence of hydrogenating environments was stated in [40]. In the study [41], round-notched wires were used for analyzing the effects of multiaxial fracture. The notch tip strain rate was analyzed to understand the HE fracture behavior in pearlitic steels [42]. 

### 1.1. Research Gaps Based on Literature Review

The literature survey revealed that the research in the HE of metallic materials is focused on analyzing the HE susceptibility in terms of (i) test results (macroscopic level): time to fracture in FIP test [24], fracture load in CERT tests [27,28,29,33,36,37,38], and geometry of the test samples [10,32,33,34,35,38], or (ii) HE damage zones (microscopic level): TTS fracture zones [16,17,18,19] and evolution of damage zones [20,21,22,23]. A key issue in this field is the selection of the most adequate round-notch geometry to be used for testing the HE susceptibility of steels. This was the topic of the study of Toribio and Ayaso [43], where the optimal notch geometry for inducing different hydrogen diffusion behaviors was determined by analyzing the distribution of stress and strain. In such distributions, different stress localizations near the notch tip were obtained in terms of the notch geometry. According to [44], strain localization appears in a nickel alloy at microstructural level because of planar slip. However, the behavior of hydrogen diffusion and hydrogen accumulation is still not revealed for such notched geometries used in CERT tests.

### 1.2. Research Questions and Intended Contribution of the Study

Considering the research gaps mentioned above, the present study addresses the following research question:

“*Can diverse scenarios be designed for testing the HE of pearlitic steels by CERT tests in terms of hydrogen microstructural damage selecting notch geometric parameters and residual stress generated by preloads*?”

The answer to this query is yes; this study tries to fill the aforesaid gap, going further in the study developed in [32]. Thus, the analysis of the hydrogen accumulation in new cases that generate residual stress and strain fields (caused by a preload) is carried out, leading to different types and locations of HAMD. This way, the effects of hydrogen at the microstructural level can be *controlled* by just varying the macroscale parameters (notch geometry and maximum preload level). Accordingly, the residual stress–strain field can be selected “*a la carte*”, causing different HE behaviors in the material tested.

### 1.3. Research Objectives

Considering the research gap and intended contribution, the objectives of the study are the following:

- To obtain diverse stress and strain fields in notched geometries, varying notch geometric parameters and residual stresses caused by a previously applied preload.
- To reveal hydrogen accumulation for estimating the high hydrogen-concentration zones where prospective hydrogen damage takes place.

To achieve these goals, two uncoupled finite element (FE) analyses were carried out in four cases of study. Thus, two different notch geometries were considered in a high-strength pearlitic steel bar under two loading sequences: (i) a constant load without preload (i.e., without residual stress) and (ii) a preload previously applied to a constant load (with a residual stress state associated). The first study deals with a numerical simulation (using an FE commercial code) of the loading sequences applied in the notched geometries for revealing the stress and strain states. From these states as input data, the second numerical simulation, dealing with the hydrogen diffusion assisted by stress and strain, is faced by using an ad hoc noncommercial FE code developed in a general-purpose mathematical software. As results, the hydrogen concentrations during a constant load test in a hydrogenating environment are estimated.

## 2. Materials and Methods

**Figure 1:**
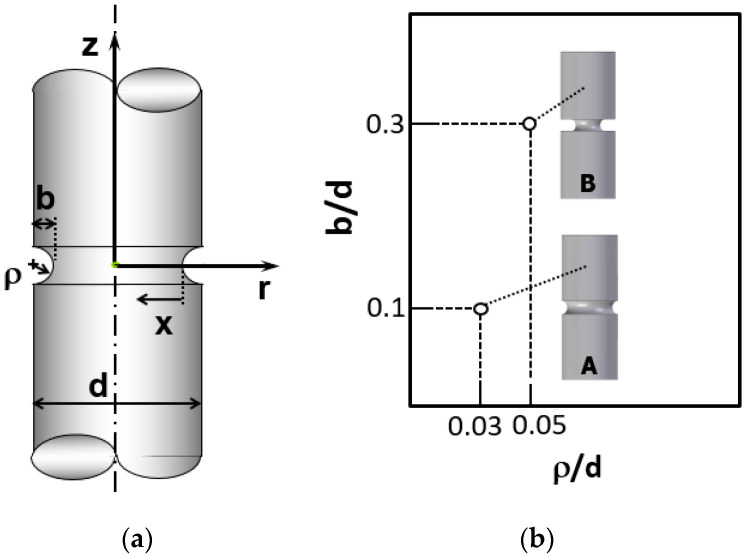
(**a**) Scheme of a notched wire including the parameters used for defining the notch geometry and (**b**) values of the dimensionless notch parameters used in notched wires A and B.

According to the study [43], stress distribution in round-notched samples is interesting for analyzing the HE of eutectoid pearlitic steels because a variation of both shape and magnitude of stress distribution can be obtained just by modifying the parameters that define the notch geometry (notch depth, *b*, and notch radius, *ρ* according to Figure 1), *x* being the radial distance from the wire surface and *d* the wire diameter.

In the study [45], it was reported the high influence of notch depth on the stress field and, hence, a similar influence on HE can be expected. Thus, in this paper, the analysis is focused on two notched wires with approximately the same notch radius but different notch depth: (i) *shallow notch* A with low depth and (ii) *deep notch* B with high depth, according to the values included in Figure 1b expressed in a dimensionless form in terms of the wire diameter *d*.

**Figure 2:**
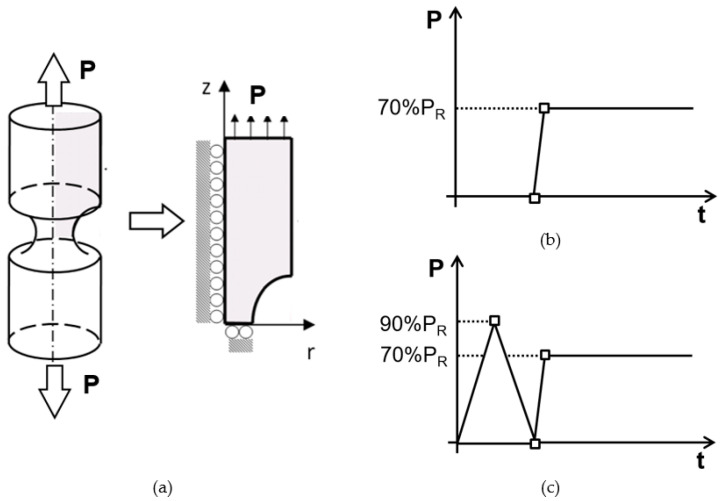
(**a**) Scheme of the reduction in the 3D geometry to an axisymmetric case including the imposed boundary conditions and load history: (**b**) without preload and (**c**) with preload.

The mechanical simulation was performed assuming a reduction in the 3D geometry to an axisymmetric 2D case that was due to the revolute symmetry of the wire. Consequently, null displacements were imposed at the symmetry axis (Figure 2a). In addition, the transversal symmetry plane at the notch tip allowed a reduction in the geometry to half, applying null displacements in the radial direction. Finally, the applied load was placed on the top of the sample. 

Two different types of loading sequence were considered: (i) a constant loading of 70% of the in-air fracture load (*P*_R_) corresponding to the in-service working loading applied to the wire in an aggressive environment (Figure 2b) and (ii) a single cycle of monotonic growing loading in air up to a maximum load of 90% *P*_R_ and later unloading; afterward, the same constant loading of 70% *P*_R_ of the un-preload case was applied to the wire in an aggressive environment (Figure 2c).The value of the in-air fracture load (notch A, *P*_R_ = 86.52 kN and notch B, *P*_R_ = 28.25 kN) was taken from [43], where similar round-notched samples were analyzed. According to [45], similar notched wires were susceptible to HE for this loading level (70% *P*_R_ = 60.56 kN for *shallow notch* A and 70% *P*_R_ = 19.77 kN for *deep notch* B) in a hydrogenating environment under an ultra-low testing rate of 3.68 × 10^−2^ mm/s for notch A and 6.02 × 10^−4^ mm/s for notch B. The 2D geometry was meshed with four-node quadrilateral elements considering different meshes up until an adequate convergence of results was achieved. 

**Figure 3:**
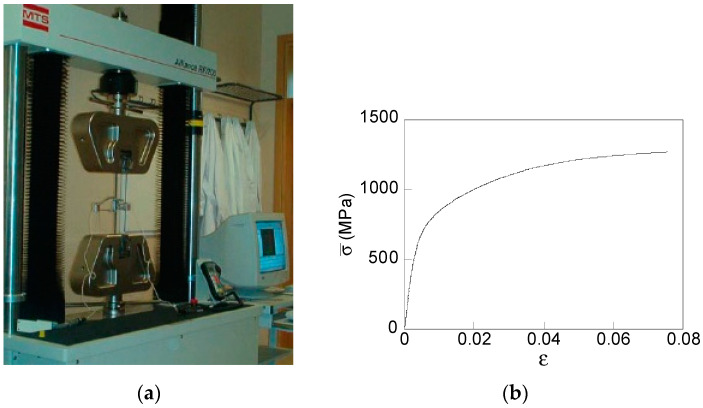
Conventional tension test: (**a**) experimental set-up and (**b**) experimental stress–strain curve of the material.

The material considered in the present work is a pearlitic steel used in a real industrial drawing chain with the following chemical composition: 0.800% C, 0.690% Mn, 0.230% Si, 0.012% P, 0.009% S, 0.004% Al, 0.265% Cr, and 0.060% V [46], provided by Spanish manufacturer *Trefilerias Quijano* (Los Corrales de Buelna, Santander, Spain). The constitutive model applied was elastoplastic solid with a von Mises yield surface, associated flow rule, and kinematic strain-hardening according to [46]. The mechanical properties of the pearlitic steel applied in the FE simulations were obtained by testing samples of 300 mm lengths of hot-rolled bars corresponding to a real commercial wire-drawing chain. Conventional tension tests up until fracture were carried out under a constant displacement rate of 2 mm/min in a universal test machine (MTS KN200, Figure 3a). From the test, the material true stress vs. true strain curve was revealed (Figure 3b). From this curve, the following mechanical properties are obtained: Young modulus, *E* = 194 GPa and yield strength, σ_Y_ = 720 Mpa.

On the other hand, the model for hydrogen diffusion assisted by stress and strain used in this work is the well-known model described in [5,6]. Briefly, it is considered that hydrogen damage appears in a material when a critical value (dependent on the stress and strain field, Equation (1)) of hydrogen concentration (*C_cr_*) is reached at certain points of the material (**x**) where a maximum hydrogen solubility (*K_S_*, dependent on the hydrostatic stress, *σ*, and equivalent plastic strains, *ε_p_*, according to Equation (2)) is achieved.

*C* (**x**, *t*) = *C*_cr_ (**x**, *σ*, *ε*) (1)

(2) $$K_{S}=K_{S\varepsilon }\left(\varepsilon_{P}\right)\exp\left(\frac{V_{H}}{RT}\sigma \right)$$

where *K_Sε_* is the hydrogen solubility dependent on plastic strains related one-to-one to equivalent plastic strains (*K_Sε_*= 1 + 4*ε_p_*, [5,13]), *V*_H_ is the molar partial volume of hydrogen, *R* is the ideal gases constant, and *T* is the absolute temperature. 

Hydrogen accumulated in these points is diffused from the wire surface (Γ) where a hydrogen concentration of equilibrium (*C_eq_*) is considered to be invariable during the time of exposure to a harsh environment.

(3) $$C_{\Gamma }=C_{eq}^{0}K_{S\varepsilon }(\varepsilon_{P,\Gamma })\exp\left(\frac{V_{H}}{RT}\sigma_{\Gamma }\right)$$

The diffusion flux (**J**) can be expressed in terms of the gradients of hydrogen concentration, gradient of hydrostatic stress, and gradient of strain-dependent hydrogen solubility (*K_Sε_*) as follows:

(4) $$J=-D(\varepsilon_{P})\left\{\nabla C-C\left[\frac{V_{H}}{RT}\nabla \sigma +\frac{\nabla K_{S\varepsilon }(\varepsilon_{P})}{K_{S\varepsilon }(\varepsilon_{P})}\right]\right\}$$

where *D* is the hydrogen diffusion coefficient.

Consequently, hydrogen diffuses toward inner points of the material with: (i) lower concentration, (ii) higher inward gradient of hydrostatic stress, and (iii) higher gradient of hydrogen solubility, which is linearly dependent on plastic strains. 

To solve this equation, a 2D axisymmetric FE approach with four-node quadrilateral elements were used, applying the weighted residual method and the Galerkin formulation. Accordingly, Equation (4) can be expressed as follows:

(5) $$\begin{array}{c} \sum_{j}\left[-\int_{V}N_{i}N_{j}dV\right]\frac{d\left[C\right]}{dt}+\sum_{j}[\int_{V}\left[D\nabla N_{i}-D\frac{v_{H}}{RT}(\nabla \sigma \nabla N_{i})N_{j}\right]dV+\vartheta \int_{S_{\mathrm{eq}}}N_{i}N_{j}dS]C_{j}\\ =J_{f}\int_{S_{f}}N_{i}dS+\vartheta C_{\mathrm{eq}}\int_{S_{\mathrm{eq}}}N_{i}dS\;\;\;\;i=1\ldots n \end{array}$$

where *N*_i_ are the shape functions, *t* is the diffusion time, *V* is the volume, *S*_f_ is the surface where a boundary condition of null flux is applied, *S*_eq_ is the surface exposed to the hydrogen equilibrium concentration, *ϑ* is a constant representing the mass exchange rate at this surface, and *n* is the total number of nodes. 

Finally, the FE system (Equation (5)) is solved by applying the algorithm proposed by Zienkiewich for ordinary differential equations [47] as follows:

(6) $$\left({\left[C\right]|}_{q}-{\left[C\right]|}_{q-1}\right)\frac{\left[M\right]+\tau \Delta t\left[K\right]}{\Delta t}+\left[K\right]{\left[C\right]|}_{q-1}=\left[F\right]$$

where the time increment Δ*t* = *t*_q_ – *t*_q−1_ and the constant *τ* are chosen to ensure the stability of this algorithm.

The simulation was performed with the following values of the parameters that have influence on hydrogen diffusion, such as temperature (*T* = 323 K), molar partial volume of hydrogen (*V*_H_ = 2 cm^3^/mol [48]), and hydrogen diffusivity in a lattice of a high-strength pearlitic steel (*D* = 6 × 10^−11^ m^2^/s [49]).

## 3. Results

To obtain different behaviors against HE in pearlitic steel wires, two variables are selected: (i) the *notch depth* and (ii) *a preload* causing a residual stress field in the wire. Thus, two uncoupled FE numerical simulations were carried out. First, a mechanical simulation of different loading sequences: (i) in-service working loading applied to the wire in aggressive environments and (ii) a preload causing residual stress and afterward, the same constant loading applied in the un-preload case. As results, the stress and strain states are revealed and, from them, a simulation of the hydrogen diffusion assisted by stress and strains is carried out for revealing the hydrogen distributions in the wire under the diverse loading conditions considered.

### 3.1. Stress and Strain States

**Figure 4:**
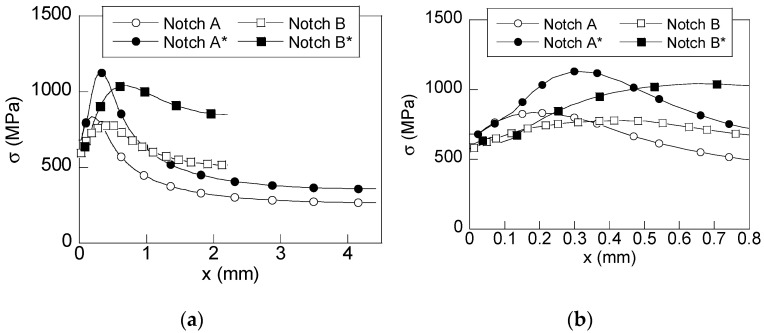
Radial distributions of hydrostatic stress generated in preloaded (filled) and un-preloaded (blank) specimens at an instant corresponding to the in-service loading (70% *P*_R_): (**a**) general distribution and (**b**) detailed view of the near-to-surface zone.

**Figure 5:**
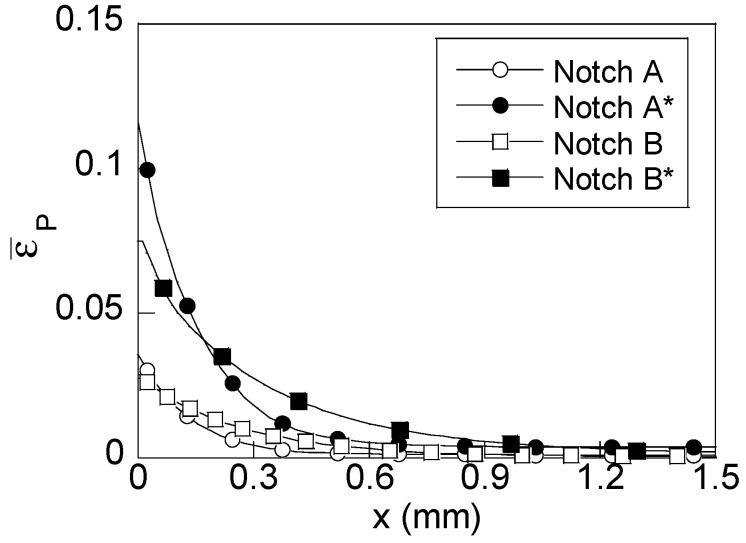
Radial distribution of equivalent plastic strain generated in preloaded (filled) and un-preloaded (blank) specimens at an instant corresponding to the in-service loading (70% *P*_R_).

Mechanical simulation allows one to obtain the wire stress and strain states during exposure to an aggressive environment. Considering the previously stated aim of this study, the main attention was paid to the variables representing the stress and strain states in the hydrogen diffusion model [5,6]. Consequently, the radial distributions of hydrostatic stress (*σ*) and equivalent plastic strain (*ε_p_*) are shown in Figure 4 and Figure 5, respectively, for both un-preloaded and preloaded notch geometries (with asterisk) at the instant corresponding to in-service loading (long time of exposure to the hydrogenating environment).

The radial distributions of the hydrostatic stress, shown in Figure 4, exhibit a similar shape: the stress is increased from the wire surface up to reaching a maximum value and, from such a point, the stress decreases up to the wire core. However, Figure 4 also shows clear differences in terms of the notch geometry and the existence (*or not*) of residual stress states caused by a previous preload. Thus, the maximum value of the hydrostatic stress is slightly higher in notched wire A (*low notch depth*) than in notched wire B (*high notch depth*). The difference is approximately 8% higher for both un-preloaded and preloaded cases. In addition, the location where the maximum value of the hydrostatic stress is reached is closer to the wire surface in the case of *shallow notch* A (*x*_max_ = 0.18 mm) than in *deep notch* B (*x*_max_ = 0.41 mm). This way, the positive inward gradient of hydrostatic stress is higher in wires with notch A than in wires with notch B. Consequently, one of the driving forces for hydrogen diffusion (the inward gradient of hydrostatic stress) can be controlled by just varying the notch depth. 

With regard to the stress state at inner points for un-preloaded specimens, notch A exhibits a sudden decrease in stress with the distance to the wire surface, reaching a stress level at the wire core around a half of the one obtained at the wire surface. However, in notch B, the stress decreases with depth more softly than in notch A, reaching at the wire core a stress level similar to that at the wire surface. This way, a more uniform stress distribution is obtained in notch B than in notch A (the ratio of the maximum to the minimum stress is around 3.1 in notch A and 1.5 in notch B). This agrees with different stress concentrations in notched wires, higher in notch A than in notch B, since the latter exhibits a higher constraint effect by the notch’s cross-sectional area. 

The effect of the preload consists in an Increment of the stress state and a displacement of the maximum of hydrostatic stress toward inner points (*x*_max_ = 0.30 mm for preloaded notch A* and, *x*_max_ = 0.65 mm for preloaded notch B*). However, neither the shape of the stress distributions nor the stress at the wire surface are significantly changed. The analysis of the near-to-surface zone (Figure 4b) reveals the effect of the compressive residual stress caused by the preload in terms of a slight reduction in the hydrostatic stress at such a zone, it being more intense in notch B. Notice that, within the zone 0 < *x* < 0.15 mm, the average inward gradient of the hydrostatic stress in the radial direction is the same for a given notch geometry. Thus, taking into account Equation (4), the hydrogen diffusion is enhanced in the geometries of type A containing a shallow notch (with and without preload) near to the wire surface, and for notch type B the effect is less intense (with lower inward gradient). For *x* > 0.15 mm, the inward gradient of hydrostatic stress in preloaded notches A* and B* is higher than that obtained in the un-preloaded cases. Accordingly, the diffusion driving force in preloaded cases acts in favor of hydrogen diffusion toward deeper points of the wire.

With regard to the plastic strain distributions shown in Figure 5, the shape is similar for all cases: plastic strain decreases with depth from the wire surface up until disappearing for a given depth. This way, the size of the plastic zone generated by loading is easily observed. However, as in the case of hydrostatic stress, key changes appear in terms of the notch geometry (notch depth) and the existence of residual stress. Thus, the equivalent plastic strain at the wire surface is slightly higher for notch A than for notch B. However, the main difference appears in the size of the plastic zone, it being higher in notch B than in notch A. Thus, the plastic strain distribution is located closer to the wire surface in notch A (0.5 mm in notch A and 0.7 mm in notch B). So, it can be considered that the plastic zone is more localized in notched geometries with low notch depths (*shallow notch* A), and plastic strains at the wire surface are higher in *shallow notch* A than in *deep notch* B. Consequently, the second driving force for hydrogen diffusion, the inward gradient of hydrogen solubility (depending one-to-one on equivalent plastic strains), is higher in samples of type A with shallow notches, it being negative in both cases. Thus, plastic strain distribution acts against hydrogen diffusion toward the inner points. This way, the importance of considering the plastic strain is revealed since the model of diffusion assisted *only* by stress does *not* consider this opposition to hydrogen diffusion caused by the negative inward gradient of plastic strains.

As previously discussed for hydrostatic stress distributions, the applied preload acts as an intensifier for the plastic strains, increasing the values of such a variable at the wire surface and also increasing the size of the plastic zone. However, the differences in terms of the notched wire geometry are the same as in the un-preloaded cases: the distribution for notch A* is more intense (higher maximum plastic strain at the wire surface) and it is more localized near the wire surface (lower size of the plastic zone) than in notch B*. This way, preload increases the inward gradients of the equivalent plastic strain in both notched geometries. Thus, the lowest inward gradient (un-preloaded notch B) is increased after a preload, thereby becoming similar to that obtained in the un-preloaded notch A. 

Consequently, several behaviors against HE are found: (i) un-preloaded notched B has the lowest opposition to hydrogen diffusion (lower inward gradient), (ii) the preloaded notch B* and the un-preloaded notch A have similar inward gradients and, finally, (iii) the highest opposition to hydrogen diffusion appears in preloaded notch A* (highest inward gradient).

### 3.2. Hydrogen Accumulation in Notched Wires

**Figure 6:**
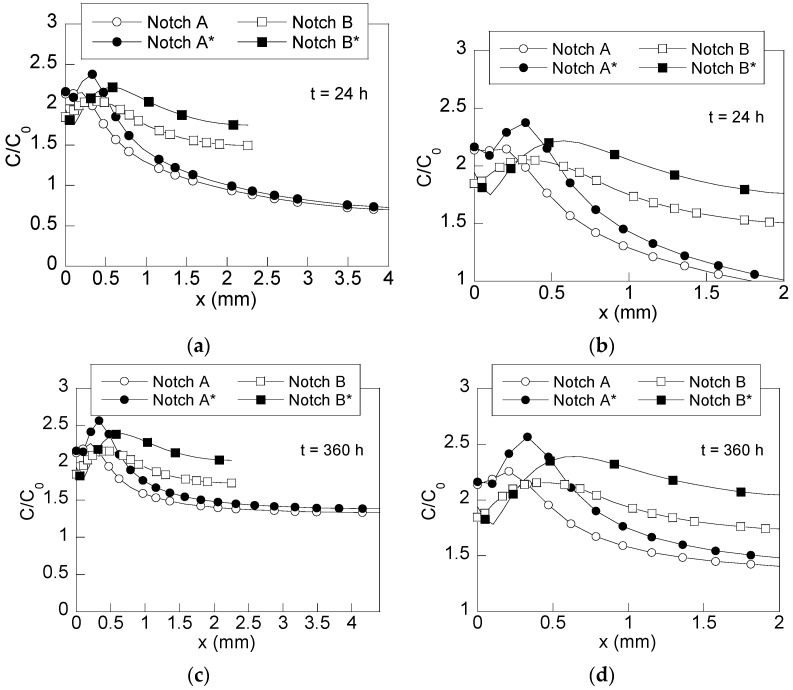
Radial distributions of hydrogen concentration in preloaded (filled) and un-preloaded (blank) specimens under in-service loading (70% *P*_R_): (i) after 24 h of exposure to the hydrogenating environment: (**a**) general view and (**b**) detailed view; (ii) after 360 h of exposure to the hydrogenating environment: (**c**) general view and (**d**) detailed view.

The simulations of the hydrogen diffusion assisted by stresses and strains reveal the radial distribution of the hydrogen concentration normalized with the hydrogen concentration of a material free of stress and strain (*C*_0_). These plots are shown in Figure 6a,b for short times of exposure to the hydrogenating environment (after 24 h) and in Figure 6c,d for the long-time ones (360 h). 

An estimation of the zones of high hydrogen concentration can be obtained from such distributions for different times of exposure to the hydrogenating environment. Thus, the prospective places where the hydrogen damage is caused by hydrogen at microstructural level are revealed. The radial distributions of hydrogen shown in Figure 6 reveal a progressive accumulation of hydrogen in the cross-section of the wire. Thus, a stationary state is reached for long times of exposure (Figure 6c), whose shape resembles the distribution of hydrostatic stress shown in Figure 4a. 

As could be expected, changes in the stress and strain states are reflected in the hydrogen accumulation profiles. This way, most of the hydrogen is accumulated near the wire surface for both notched geometries. However, in the case of notch A, this effect is more intense, reaching higher concentrations over a narrower zone (the depth of the maximum of the hydrostatic stress, *x* = 0.2 mm) than the one corresponding to notch B (*x* = 0.4 mm). On the other hand, the effect of the residual stress and strain states caused by a preload is given by two factors located at the notch tip surroundings: (i) a slight increment of the maximum hydrogen concentration (around a 15%) and (ii) a displacement of the maximum hydrogen concentration toward inner points (*x* = 0.33 mm for notch A* and *x* = 0.65 mm for notch B*), in a similar way previously observed in hydrostatic stress distributions (cf. Figure 4a). 

In addition, the effects of compressive residual stress near the wire surface caused after applying the preload are revealed. Thus, a reduction in the hydrogen concentration appears at the notch-tip surroundings (visible even for early times of exposure). This effect is more intense in *deep notch* B than in *shallow notch* A. 

This way, four different behaviors against hydrogen diffusion can be obtained just by varying one macroscopic geometrical variable: the notch depth. Thus, the location of the highest hydrogen concentration, which can be linked with the size of the hydrogen damage zone, can be placed at 0.20 mm, 0.30 mm, 0.40 mm, or 0.65 mm from the wire surface with similar damage levels, i.e., with similar hydrogen concentrations (the relative difference is always lower than 15%). Thus, different patterns of hydrogen migration can be obtained as a function of the parameters analyzed in this study, i.e., the notch geometry and the applied preload. Therefore, taking into account the heavy stress concentration reached near the notch tip and the plastic strains distributed through a narrow zone, the use of *shallow notch* A allows the transport of hydrogen from the wire surface toward a zone placed very close to the notch tip. However, by using *deep notch* B, the maximum stress is placed deeper, and the plastic strains are distributed through a wider zone. Both effects allow hydrogen to diffuse from the wire surface toward deeper points than in the previous case. The effects of compressive residual stress caused after preloading modify the hydrogen migration within the wire. Thus, for the particular case of *preloaded shallow notch* A*, the distribution of stress is similar to the un-preloaded case (notch A), but the maximum stress is shifted toward inner points, allowing hydrogen to reach a deeper zone for causing hydrogen microdamage in the material. For the case of *preloaded deep notch* B*, the applied preload causes a similar effect; consequently, hydrogen is transported from the wire surface toward the deepest points in the sample.

Most of the hydrogen damage is reached for similar times of exposure to the hydrogen environment because of the competing effects on hydrogen diffusion of the two driving forces: the high positive inward gradient (higher in *shallow notch* A) is balanced with the high negative inward gradient of equivalent plastic strains. Therefore, similar times of exposure are needed for reaching high hydrogen concentrations at the wire surface surroundings for all considered geometries. As can be observed comparing Figure 6a,c, a 95% of the maximum concentration obtained after 15 days (Figure 6c) is reached after 24 h of hydrogenation (Figure 6a) in all cases. 

## 4. Conclusions

Notch depth is a key parameter affecting the radial distributions of stress and plastic strain. Accordingly, different behaviors in hydrogen diffusion and diverse hydrogen accumulation in a steel notched wire can be obtained. This way, according to the obtained results, four different scenarios are established for estimating the hydrogen embrittlement (HE) susceptibility of high-strength pearlitic steel rods just by combining two notch depths and the residual stress and plastic strain caused by a preload.

Thus, un-preloaded *shallow notches* (*without* residual stress) are appropriated for obtaining hydrogen damage placed close to the wire surface. This is due to the fact that stress and plastic strain fields are heavily localized close to the wire surface. On the other hand, un-preloaded *deep notches* (*without* residual stress) are suitable for placing hydrogen damage at deeper points because stress and strain are more uniformly distributed. Results reveal the key role of plastic strains in hydrogen diffusion because two competitive effects were observed. On one hand, the positive inward gradient of hydrostatic stress enhances the hydrogen diffusion and, on the other hand, the negative inward gradient of equivalent plastic strain acts against hydrogen diffusion. Results revealed that both effects are higher in *shallow notches*.

A way of obtaining deeper hydrogen-damage zones is to apply a *preload* before testing the HE susceptibility of a material. According to the obtained results, the compressive residual stresses and plastic strains generated near the wire surface after applying a preload cause a displacement of the maximum hydrostatic stress and hydrogen concentration toward inner points of the wire. As a result, deeper hydrogen-damage zones can be obtained for both *shallow notches* and *deep notches* without significantly modifying the maximum hydrogen concentrations or the time of exposure to the hydrogenating environment.

This way, diverse hydrogen micro-damage scenarios can be selected “*a la carte*” for estimating the HE susceptibility of a material. Thus, properly modifying the notch geometry and the residual stress produced by a preload, different stress and strain fields can be obtained. Therefore, the hydrogen diffusion and hydrogen accumulation at the prospective hydrogen-damage zones in any metallic material can be controlled using the *quite general* procedure proposed in this paper on the basis of using notched samples with and without a pre-loading history.

## Data Availability

Not applicable.
